# Blood urea nitrogen concentration is associated with severe abdominal aortic calcification in adults: a cross-sectional investigation

**DOI:** 10.1038/s41598-023-47109-5

**Published:** 2023-11-13

**Authors:** Kun Xue, Shanshan Xing

**Affiliations:** Shandong University of Traditional Chinese Medicine, Jinan, Shandong China

**Keywords:** Cardiology, Health care, Health occupations, Medical research

## Abstract

The purpose of this research is to examine the correlation between blood urea nitrogen (BUN) and severe abdominal aortic calcification (AAC) among American adults aged 40 years and older. A total of 2757 participants in the NHANES from 2013 to 2014 were included in the final analysis. BUN was measured by means of the enzymatic conductivity rate method. AAC scores were quantified by the Kauppila scoring system, and severe AAC was defined as an AAC score ≥ 6. Multivariable logistic regression and restricted cubic splines were used in the analyses. In the multivariable logistic regression model, the highest BUN level (log 2-transformed) was associated with an increased risk of severe AAC [odds ratio (OR) = 1.77, 95% CI 1.17, 2.71]. The restricted cubic spline plot displayed a reverse l-shaped association between BUN (log2-transformed) and severe AAC (p for nonlinearity < 0.001). In addition,the interactions of BUN were not discover. In general, there is a positive correlation between BUN and the risk of severe AAC.

## Introduction

Utilizing standard lateral lumbar radiographs to evaluate abdominal aortic calcification (AAC), it is identified as a marker for asymptomatic atherosclerotic conditions and functions as an independent forecaster for ensuing vascular diseases and mortalities^1^. According to previous study, atherosclerosis, a disease of the large arteries, is the primary cause of heart disease and stroke^2,3^. It is the fundamental reason for approximately half of all fatalities^4^. Nonetheless, there is presently no effective therapy for serious AAC. To assess personalized cardiovascular risk, recognizing atherosclerosis in its subclinical phase is crucial^5^.

Blood urea nitrogen (BUN) is a laboratory test that measures the amount of nitrogen in the blood that comes from urea, a waste product formed by the liver^6^. Elevated BUN levels are often seen in individuals with chronic kidney disease (CKD), which is frequently associated with cardiovascular disease (CVD)^7,8^. Numerous studies indicate that the BUN/creatinine ratio, which functions independently of both BUN and creatinine, is a well-established predictor of adverse outcomes in patients with CVD^9,10^. In addition, some research suggests that in Chinese communities, a higher BUN level may be linked to a higher risk of incident coronary artery disease(CAD)^11^. Moreover, most previous studies were investigated among patients with heart failure^12^, acute coronary syndrome^13^, or stroke^14^. However, no cross-sectional study has examined the relationship between BUN and severe AAC among a general population.

Hence, we carried out this research to examine the correlations between BUN and severe AAC, utilizing data from the National Health and Nutritional Examination Survey (NHANES) for the years 2013–2014.

## Methods

### Ethics statement

The studies involving human participants were ethically approved by The National Center for Health Statistics (NCHS) Ethics Review Board. Prior to data collection, all participants provided written informed consent. All methods were conducted in accordance with the relevant guidelines and regulations of the NCHS Institutional Review Board.

### Study population

NHANES is a survey that employs a complex multistage probability sampling design and oversamples minority populations to ensure accurate representation of the US population. Individual participants are assigned appropriate sampling weights, and continuous NHANES data have been accessible since 1999, released every 2 years^15^.

**Figure 1:**
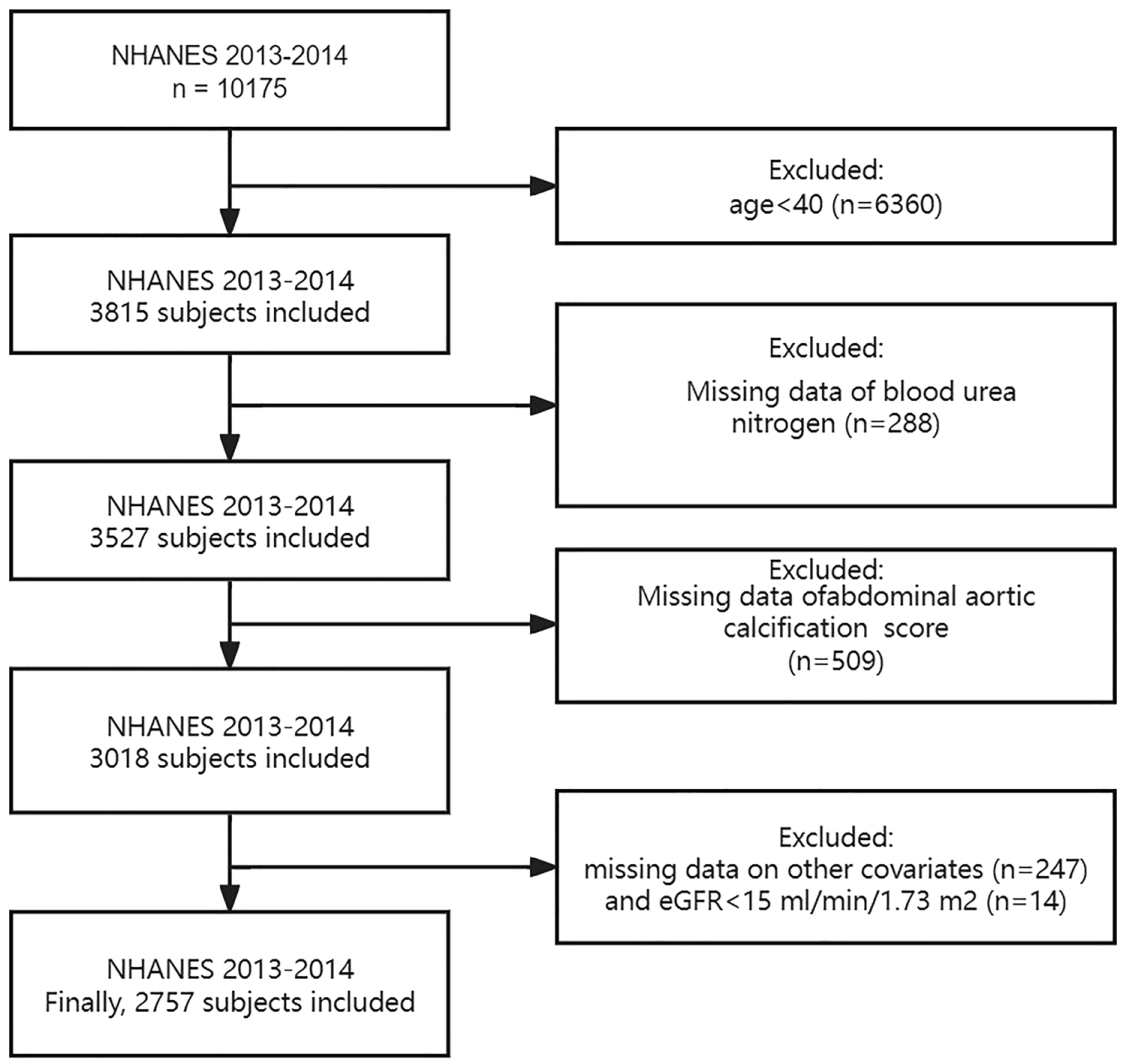
Flowchart of participants included in the analysis.

Our investigation of the potential correlation between BUN and severe AAC was grounded on data from the 2013–2014 NHANES cycle. This is because only this specific cycle contains information on both BUN and AAC scores. Furthermore, individuals under the age of 40 were not considered in our analysis because that AAC scores were determined through dual-energy X-ray absorptiometry (DXA) scans, which were not conducted on participants below this age during the relevant NHANES cycle in 2013–2014. Our analysis was limited to individuals aged 40 years or older who had complete data on both blood urea nitrogen and abdominal aortic calcification (AAC) scores. Initially, a total of 10,175 participants were included in the study. However, after excluding individuals under the age of 40 (n = 6360), those with incomplete BUN data (n = 288), missing AAC scores (n = 509), or incomplete data on other covariates (n = 247) and estimated glomerular filtration rate (eGFR) < 15 ml/min/1.73 m^2^ (n = 14), such as demographics and blood biochemical indexes, the final sample size for analysis was reduced to 2757 participants (Fig. 1).

The protocol for NHANES received clearance and endorsement from the National Center for Health Statistics’ Ethics Review Board. All participants provided written informed consent before any data collection commenced.

### Exposure and outcome definitions

The AAC score was determined by evaluating lateral lumbar spine images acquired from DXA (Densitometer Discovery A, Hologic, Marlborough, MA, USA), executed by trained NHANES affiliated personnel during a single assessment in a mobile examination center (MEC). Utilizing the Kauppila scoring method, which has been extensively cited and employed for evaluating calcified vessel severity, professional technologists assigned a total AAC score for each participant with scores ranging from 0 to 24^16^. Previous research has also identified severe AAC as an important outcome measure, defined by a total AAC score ≥ 6, indicating the presence of significant aortic calcification lesions^1,17^.

Blood urea nitrogen samples should to be procured from non-hemolyzed specimens, and there is no need for fasting. If samples are stored beyond 24 h, they should be preserved at temperatures between − 15 °C and − 20 °C, with only one thawing instance permitted. Inadequate samples may consist of insufficient volume (< 0.6 mL), hemolysis, incorrect labeling, or extended exposure of serum or plasma to cells.

### Covariates

A standardized survey was utilized to gather demographic data, encompassing respondents’ age (< 60 and ≥ 60), race (Mexican American, Other Hispanic, non-Hispanic White, non-Hispanic Black, and other), educational level (Less Than 9th Grade, 9–11th Grade, High School Grad/GED, Some College or AA degree, and College Graduate or above), body mass index (BMI),alcohol use (drinker, nondrinker),smoking status (yes/no), alanine aminotransferase (ALT), aspartate aminotransferase (AST), serum vitamin B12, eGFR, hypertension(yes/no), diabetes status(yes/no) and income-to-poverty ratio (PIR). Individuals who reported consuming a minimum of 12 alcoholic beverages in any given year were categorized as drinkers. Individuals who had consumed a minimum of 100 cigarettes throughout their lives were categorized as smokers. The criteria for diabetes encompassed the use of hypoglycemic medications or a medical diagnosis, in addition to satisfying specific laboratory benchmarks, such as a hemoglobin A1c concentration of  ≥ 6.5% and/or a fasting plasma glucose concentration of ≥ 126 mg/dL^18^. The definition of hypertension included taking medications for high blood pressure, having a medical diagnosis of hypertension, or having average systolic blood pressure readings greater than or equal to 140 mmHg, and/or diastolic blood pressure readings greater than or equal to 90 mmHg^19^. Using BMI cutoff points, subjects were divided into three groups: underweight (< 18.5 kg/m^2^), normal weight (18.5–25 kg/m^2^), over weight (25–30 kg/m^2^), and obesity (≥ 30 kg/m^2^). The chronic kidney disease epidemiology collaboration creatinine equation^20^ was used to determine the eGFR. The measurement methodologies for the research variables are fully described on the publically available website: www.cdc.gov/nchs/nhanes/.

### Statistical analysis

In light of the intricate, multistage, probability sampling approach used in the NHANES survey, oversampling occurred for representative participants of specific civilian, noninstitutionalized US subpopulations. Consequently, we incorporated sample weights that had been established in the NHANES analysis. To generate unbiased national estimates, the study utilized the sample weight.

The categorical data were described by frequency (n) and percentage (%), and continuous data were described by the mean ± standard deviation (SD). BUN was skewed distributed and log 2-transformed to improve the normality of the data. We divided the BUN(log 2-transformed) concentration into four categories and study participants were categorized, based on quartiles of BUN. In relation to BUN quartiles, general attributes were compared utilizing the Wilcoxon rank-sum test for continuous variables, and the chi-square test for categorical variables. Three separate multivariate logistic regression models were applied to examine the independent connection between BUN and severe AAC. Model 1 was the unadjusted version. Model 2 incorporated age, sex, and ethnicity. Model 3 extended upon Model 2 by adding body mass index (BMI), education level, alcohol consumption, smoking status, diabetes, ALT, AST, serum vitamin B12, PIR, eGFR and hypertension as additional variables. Subgroup analysis categorized by age, gender, hypertension, smoking status, and diabetes, was conducted using stratified multivariate regression analysis. Additionally, an interaction term was introduced to examine the heterogeneity in the associations among the subgroups. The nonlinear association between BUN and severe AAC was analyzed using restricted cubic splines (RCS) to address potential nonlinearities.

All analyses were conducted using table one and the foreign package from R (version 4.2.2) and GraphPad Prism (version 9.0.0). Statistical significance was determined by two-sided p values of less than 0.05.

## Results

### Baseline characteristics of the enrolled participants

The essential traits of the research participants are displayed in Table 1. Of the participants, 303 (9.6%) had severe AAC. Participants with severe AAC were older, more likely to be female, white, and smokers than those without the condition. In addition, there was a greater frequency of diabetes, hypertension, elevated serum vitamin B12 levels, elevated BUN, and decreased eGFR in these individuals.

**Table 1:** Weighted characteristics of participants based on abdominal aortic calcification.

Characteristic	Non-severe AAC, N = 2454 (90%)	Severe AAC, N = 303 (9.6%)	P value
Age group			** < 0.001**
< 60	1431 (64%)	41 (14%)	
≥ 60	1023 (36%)	262 (86%)	
Sex			0.3
Female	1266 (51%)	159 (56%)	
Male	1188 (49%)	144 (44%)	
Race			**0.030**
Mexican American	314 (6.72%)	25 (3.97%)	
Other Hispanic	237 (4.49%)	14 (2.53%)	
Non-Hispanic White	1068.00 (71.69%)	198 (80.66%)	
Non-Hispanic Black	482 (9.82%)	40 (6.64%)	
Other/multiracial	353 (7.28%)	26 (6.20%)	
Education level			**0.021**
Less than 9th grade	211 (4.49%)	29 (6.53%)	
9–11th grade	313 (9.48%)	49 (14.47%)	
High school grad/GED	551 (21.68%)	74 (24.48%)	
Some college or AA degree	714 (30.44%)	81 (30.54%)	
College graduate or above	665 (33.92%)	70 (23.98%)	
Alcohol use			0.10
Drinker	1676 (75.56%)	212 (71.09%)	
Non-drinker	778 (24.44%)	91 (28.91%)	
BMI(kg/m^2^)			**0.002**
Underweight	52 (1.73%)	7 (1.85%)	
Normal weight	652 (25.46%)	92 (28.00%)	
Overweight	867 (36.44%)	135 (46.99%)	
Obesity	883 (36.36%)	69 (23.16%)	
Diabetes, %	501 (16%)	108 (33%)	** < 0.001**
Hypertension, %	1245 (48%)	239 (77%)	** < 0.001**
Smoker, %	1112 (45%)	184 (61%)	** < 0.001**
PIR	3.22 ± (1.63)	2.78 ± (1.50)	**0.009**
Serum vitamin B12 (pg/ml)	648.33 ± (764.00)	726.66 ± (624.61)	**0.015**
ALT (U/l)	25.02 ± (15.28)	23.54 ± (30.14)	**0.022**
AST (U/l)	25.52 ± (14.53)	26.02 ± (20.97)	0.6
BUN(mg/dl)	13.8 ± (5.1)	17.3 ± (7.6)	** < 0.001**
eGFR (mL/min/1.73 m2)	82 ± (19)	69 ± (22)	** < 0.001**

Mean ± SD was for continuous variables. n % was for categorical variables.

*PIR* poverty income ratio, *BMI* body mass index, *AAC* abdominal aortic calcification, *ALT* alanine aminotransferase, *AST* aspartate aminotransferase, *BUN* blood urea nitrogen, *eGFR* estimated glomerular filtration rate.

Significant values are in bold.

### Association of blood urea nitrogen with severe abdominal aortic calcification

We used three logistic regression models to show the relationship between BUN and severe AAC in Table 2: The BUN index was categorized based on quartiles, and when comparing participants in the lowest quartile to those in the highest quartile, higher odds for extensive AAC were found across all three models (Model 1: OR = 3.54, 95% CI 2.53–5.03; Model 2: OR = 1.92, 95% CI 1.33–2.82; Model 3: OR = 1.77, 95% CI 1.17–2.71; all p-value < 0.05).In the fully adjusted model (model 3), we found that compared with the first quartile (Q1),the prevalence of severe AAC in the highest quartile group (Q4) increased by 77%.

**Table 2:** Multivariate logistic regression models of blood urea nitrogen with severe abdominal aortic calcification.

Variable	Model 1			Model 2			Model 3		
	OR	95% CI	P value	OR	95% CI	P value	OR	95% CI	P value
BUN									
Q1	Ref			Ref			Ref		
Q2	1.24	(0.79, 1.91)	0.343	1.17	(0.73, 1.86)	0.515	1.31	(0.81, 2.11)	0.271
Q3	1.42	(0.95, 2.11)	0.083	1.08	(0.71, 1.65)	0.719	1.08	(0.70, 1.68)	0.728
Q4 P for trend	3.54	(2.53, 5.03) ** < 0.001**	** < 0.001**	1.92	(1.33, 2.82) ** < 0.001**	** < 0.001**	1.77	(1.17, 2.71) **0.010**	**0.007**

Model 1, no covariate was adjusted; Model 2, age group, sex, and race were adjusted; Model 3, age group, sex, race, education level,PIR, BMI,alcohol use, smoking status, hypertension, diabetes, serum vitamin B12, ALT, eGFR and AST were adjusted.

*OR* odds ratio, *CI* confidence interval, *AAC* abdominal aortic calcification, *BUN* blood urea nitrogen, *PIR* poverty income ratio, *BMI* body mass index, *ALT* alanine aminotransferase, *AST* aspartate aminotransferase, *eGFR* estimated glomerular filtration rate.

Significant values are in bold.

**Figure 2:**
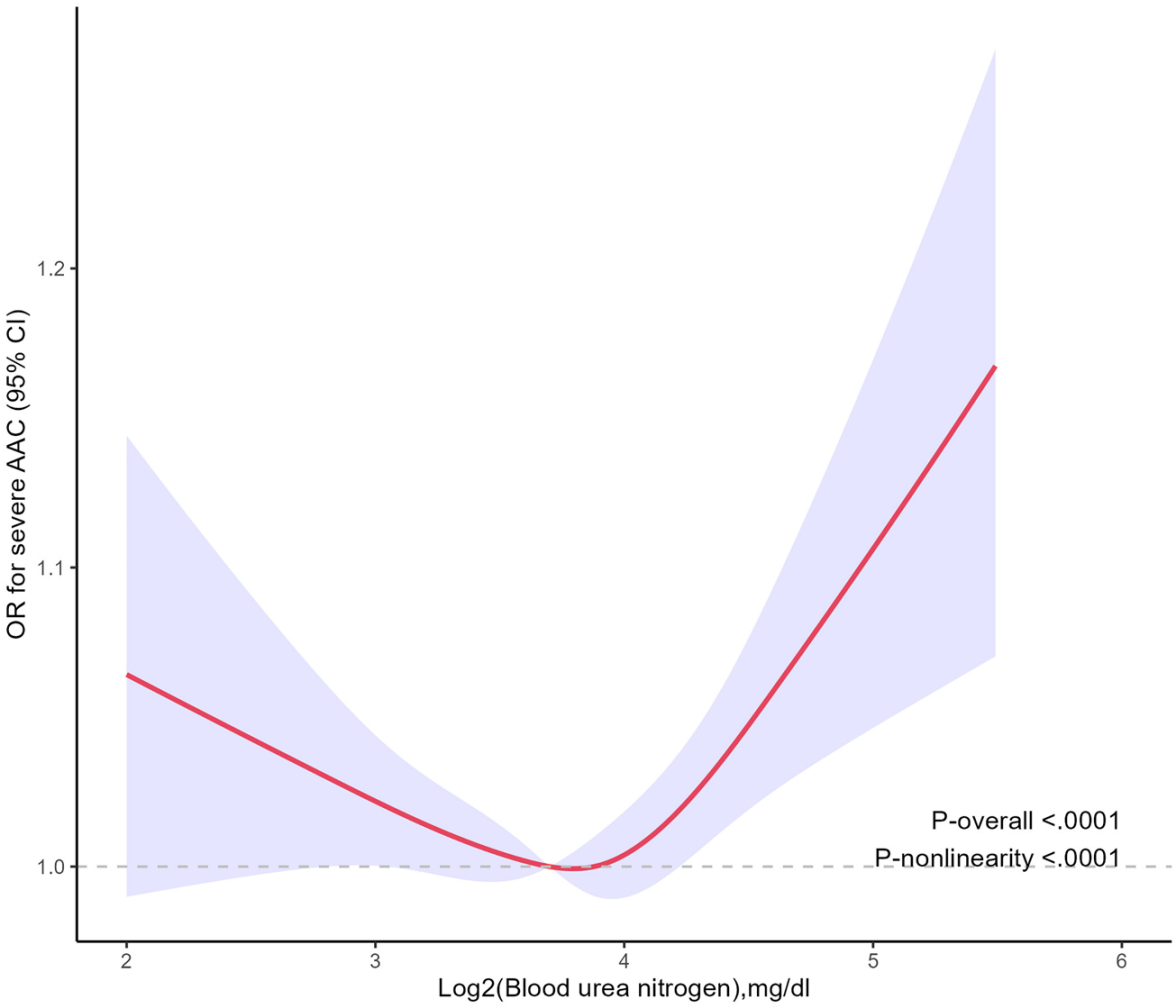
Association between BUN level and severe AAC. Adjusted for age group, sex, race, education level, PIR, BMI, alcohol use, smoking status, hypertension, diabetes, serum vitamin B12, ALT, eGFR and AST. The solid and shaded areas represent the odds ratios and corresponding 95% confidence intervals. *OR* odds ratio, *AAC* abdominal aortic calcification, *BUN* blood urea nitrogen, *PIR* poverty income ratio, *BMI* body mass index, *ALT* alanine aminotransferase, *AST* aspartate aminotransferase, *eGFR* estimated glomerular filtration rate.

As shown in Fig. 2, the restricted cubic spline plot displayed a reverse l-shaped association between blood urea nitrogen (log 2-transformed) and severe AAC (p for nonlinearity < 0.001). We further conducted a threshold effect analysis of the association between BUN (log 2-transformed) and severe AAC (Table 3) after adjustment for multiple potential covariates. We fit the l-line logistic regression model and 2-piecewise logistic regression model to test the relationship between BUN and severe AAC. The results indicated that the 2-piecewise logistic regression model was superior to the l-line logistic regression model for fitting the association between blood urea nitrogen and severe AAC (p for the log likelihood ratio test < 0.05). We identified an inflection point of 3.79 mg/dl for log 2-transformed blood urea nitrogen. When blood urea nitrogen was > 3.79 mg/dl (Log2- transformed), each onefold increase in blood urea nitrogen was significantly associated with a 87% increase in the prevalence of severe AAC (OR 1.87, 95% CI 1.13, 3.09).

**Table 3:** Threshold effect analysis of blood urea nitrogen on severe AAC using piecewise binary logistic regression models.

Outcomes	OR	95% CI	P value, log likelihood ratio tests
BUN ≤ 3.79 mg/dl	0.76	0.43, 1.34	0.327, 0.028
BUN > 3.79 mg/dl	1.87	1.13, 3.09	0.015

Adjusted for age group, sex, race, education level, PIR, BMI, alcohol use, smoking status, hypertension, diabetes, serum vitamin B12, ALT, eGFR and AST.

*OR* odds ratio, *CI* confidence interval, *AAC* abdominal aortic calcification, *BUN* blood urea nitrogen, *PIR* poverty income ratio, *BMI* body mass index, *ALT* alanine aminotransferase, *AST* aspartate aminotransferase, *eGFR* estimated glomerular filtration rate.

### Subgroup analyses

**Figure 3:**
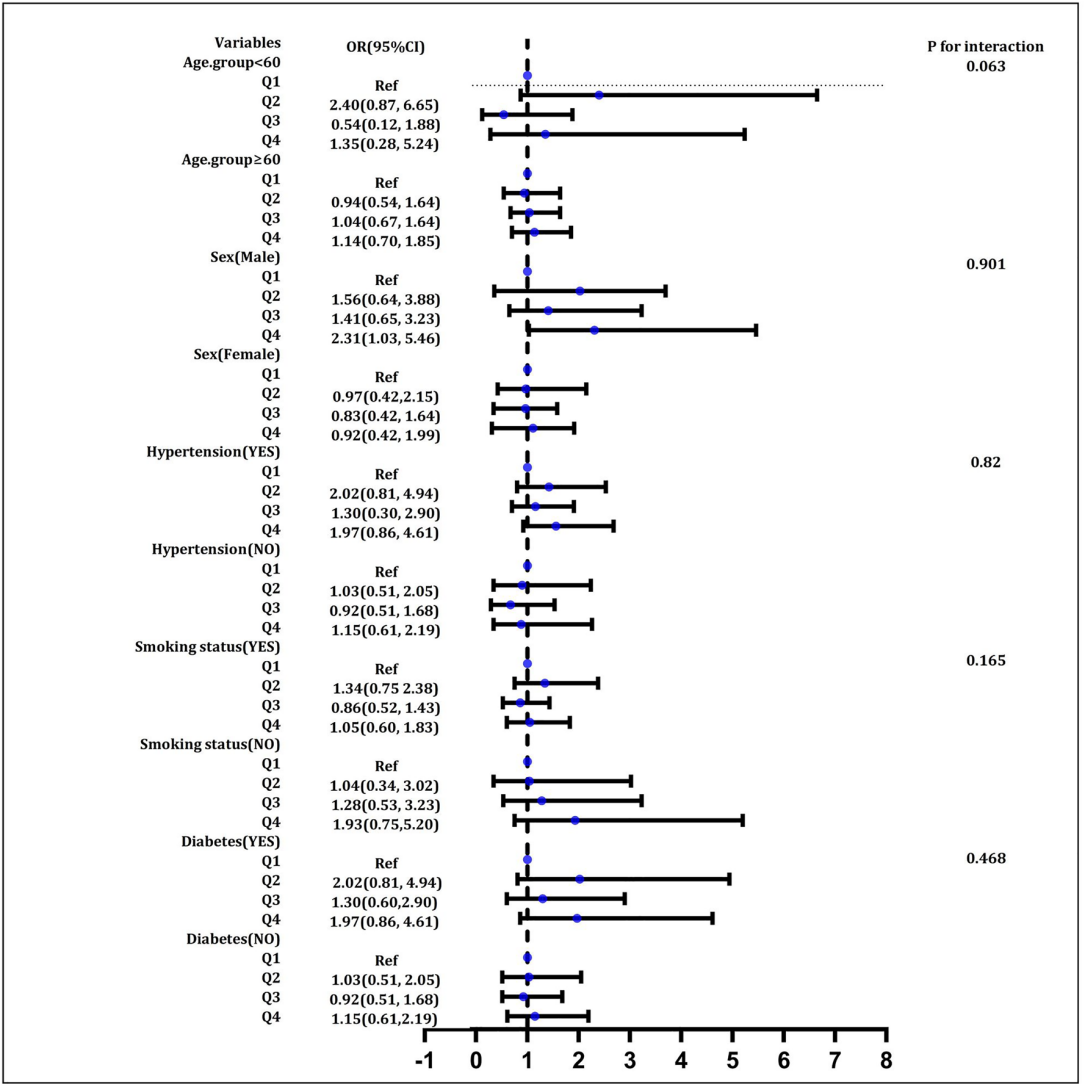
Subgroup analysis for the association between BUN and severe AAC.

Subgroup analyses stratified by age, sex, smoking status, hypertension, and diabetes was subsequently performed and are illustrated in Fig. 3. We discovered that in all multivariate logistic regression models, the relationship between BUN and severe AAC in females remained statistically significant. No notable interactions have come to our attention (all P for interaction > 0.05).

## Discussion

Within a nationally representative US cohort, we identified an association between BUN levels and the risk of severe AAC. A notable positive relationship was found between overall BUN concentrations and severe AAC. In this study, the smooth fitting curve between BUN and severe AAC was reverse L-shaped and the inflection point was > 3.79 mg/dL (Log2- transformed).

There is substantial clinical significance to these findings. First, a subclinical characteristic of CVD is AAC. Our findings offer a fresh method for assessing the risk of severe AAC in the general population. We suggested that people with high BUN levels would benefit from regular cardiac health monitoring to lower their chance of developing CVD in the future. Second, when assessing the risk of CVD in middle-aged and older persons, particularly in females, BUN level should be included in addition to conventional CVD risk factors.

A prevalent functional indicator of renal function is blood urea nitrogen. A growing body of research indicates that BUN is a reasonably common routine test that can accurately identify high-risk individuals with acute coronary syndrome (ACS) so that any unfavorable vascular events are closely monitored^20^. Moreover, earlier research has shown that eGFR had little bearing on the relationships found between BUN level and coronary heart disease^11^ or other cardiovascular outcomes^13^. More significantly, logistic regression analysis demonstrated that, once the effects of other variables (such as age, gender, comorbidities, cardiac enzymes, etc.) on the outcome were eliminated, patients whose BUN levels were higher than our observed cut-off values were nearly 20 times more likely to suffer adverse cardiovascular events that resulted in mortality^21–23^. Prior research revealed that, after adjusting for eGFR levels, a higher BUN level was linked to a 17% increased risk of coronary heart disease^11^. Consistent with previous research, we discovered noteworthy correlations between elevated BUN and the likelihood of severe AAC, even after accounting for eGFR. These conclusions held true even after excluding individuals whose eGFR was less than 15 ml/min/1.73 m2. In order to confirm our results and determine the appropriate normal range of BUN for the prevention of AAC, bigger sample sizes will be needed in future research.

While BUN is acknowledged as a possible risk factor for cardiovascular disease, the specific mechanisms have yet to be fully understood. One plausible biological pathway that connects increased BUN levels and AAC could involve disruptions in glucose balance^24,25^. BUN not only indicates kidney performance but is also associated with the stimulation of neurohormones^26^. Increased BUN reabsorption may be the outcome of elevated vasopressin levels in patients with heart failure. It might be connected to atherosclerosis because oxidative stress damages the arterial wall and causes myocardial ischemia or infarction in the end^27^. In addition, the enhancement in urea reabsorption can be attributed to the stimulation of the sympathetic nervous system and the activation of the renin–angiotensin–aldosterone system which is known to have a connection with cardiovascular risk^28^.

Our investigation possesses numerous advantages. First, it utilizes data from NHANES, an extensive, countrywide, population-based sample gathered through a uniform methodology. Furthermore, we accounted for potential confounding factors, with the choice of these variables primarily derived from earlier research evaluating the association between AAC and other relevant exposures, thus ensuring the dependability of our findings.

Nonetheless, certain limitations must be acknowledged. Although we adjusted for some potential covariates, it was not possible to entirely eliminate the impact of other potential confounders, such as diuretics, hormones, and medication usage, which might affect calcification. Moreover, our sample solely consisted of participants from one country, making it less applicable to the global population with diverse ethnic backgrounds. Last, the subject reported disease history could be subject to recall errors.

## Conclusions

In general, there is a positive correlation between BUN and the risk of severe AAC. In addition to traditional CVD risk factors, BUN level should be taken into consideration. Such findings require further prospective studies to provide more evidence.

## Data Availability

The datasets generated and analyzed during the current study are available in the National Health and Nutrition Examination Survey (NHANES), www.cdc.gov/nchs/nhanes/.
